# Neutrophil extracellular traps in gynecological disease: pathogenic mechanisms and therapeutic opportunities

**DOI:** 10.3389/fmed.2026.1710628

**Published:** 2026-02-05

**Authors:** Meiling Wu, Jingxin Ding, Xuyin Zhang

**Affiliations:** Obstetrics & Gynecology Hospital of Fudan University, Shanghai Key Lab of Reproduction and Development, Shanghai Key Lab of Female Reproductive Endocrine Related Diseases, Shanghai, China

**Keywords:** neutrophil extracellular traps (NETs), tumor immune microenvironment, gynecologic cancers, endometriosis, reproductive endocrine disorders, chronic inflammation

## Abstract

Gynecologic disorders, including infections, sterile inflammatory diseases, endocrine abnormalities, and malignancies, share a common signature of dysregulated immunity within a uniquely hormone-responsive reproductive tract. Neutrophil extracellular traps (NETs) are increasingly recognized as central effectors at this interface of innate immunity, endocrine signaling, tissue remodeling, and thrombosis. In this review, we first outline the mechanistic basis of NET formation and emphasize how the cyclical anatomy, fluctuating sex hormones, and regional microbiota of the female reproductive tract shape NET induction, localization, and clearance. We then synthesize evidence across disease spectra. In infectious conditions such as pelvic inflammatory disease, genital tuberculosis, and vaginal dysbiosis, NETs confine pathogens but also drive epithelial injury, fibrosis, and infertility. In sterile inflammatory and endocrine-related disorders, including endometriosis, polycystic ovary syndrome, premature ovarian insufficiency, and primary dysmenorrhea, NET-associated oxidative stress, inflammasome activation, and profibrotic signaling link hormonal and metabolic imbalance to chronic pain and organ dysfunction. In gynecologic cancers, NETs promote tumor cell adhesion, invasion, immune escape, and thromboembolic complications within hormone-conditioned microenvironments, while circulating and tissue NET markers, as well as NET-related gene and lncRNA signatures, hold diagnostic and prognostic value. Finally, we discuss how biomaterial-based strategies in vaginal reconstruction exploit antimicrobial NET functions yet risk excessive fibrosis if NETs are not tightly controlled. Across these contexts, we highlight an emerging NET–sex hormone axis and propose endocrine-aware, biomarker-guided strategies that combine NET-targeting agents with hormonal and microbiome-based interventions to achieve more precise diagnosis, risk stratification, and therapy for gynecologic diseases.

## Highlights

- The female reproductive tract provides a cyclic, hormone-responsive, and microbiota-rich niche that uniquely modulates NET induction, localization, and clearance.
- Infectious gynecologic diseases and vaginal dysbiosis converge on exaggerated NETosis, which couples pathogen control to epithelial damage, fibrosis, adhesions, and infertility.
- In endometriosis and reproductive endocrine disorders such as PCOS, POI, and primary dysmenorrhea, NET-driven oxidative stress, inflammasome activation, and profibrotic signaling bridge hormonal or metabolic imbalance with chronic pain and ovarian failure.
- In cervical, ovarian, endometrial cancer, and gestational trophoblastic neoplasia, NETs foster metastatic seeding, immunosuppression, and thrombosis, while NET-related circulating biomarkers and gene signatures refine prognosis and therapeutic stratification.
- Biomaterial-based vaginal reconstruction and future NET-targeted therapies must be designed in an endocrine-aware manner, balancing antimicrobial benefits with the risk of fibrosis and integrating NET inhibition with hormonal and microbiome-directed interventions.

## Introduction

Gynecological disorders, including infections, chronic sterile inflammation, endocrine abnormalities, and malignant tumors, adversely affect women’s reproductive function, fertility, and overall health (1, 2). Regardless of their etiology, these diseases often feature dysregulated immune responses and persistent inflammation in the local tissue environment. The female reproductive tract represents a uniquely specialized and dynamic immunological environment (3, 4). Anatomical structures, such as the cervix with alternating open and closed barrier states and the uterine cavity undergoing cyclical remodeling, generate spatially heterogeneous immune niches that constrain leukocyte trafficking and local signaling (5). Superimposed on this architecture are recurrent cycles of tissue breakdown and repair associated with ovulation and menstruation, producing transient inflammatory microenvironments that require tightly regulated immune responses (6, 7). Additionally, cyclical fluctuations of sex hormones, particularly estrogen and progesterone, modulate immune cell recruitment, activation, and effector functions, while hormone-dependent gynecologic disorders, including endometriosis and certain hormone-related tumors, further amplify local endocrine-driven immunoregulation (7). Recent meta-analysis demonstrates that concentrations of multiple immunomodulatory mediators in the cervicovaginal tract (chemokines, interleukins, MMPs, antimicrobial peptides) vary significantly throughout the menstrual cycle, supporting that cyclic hormonal changes reshape the local immune microenvironment in a predictable manner. Moreover, endogenous progesterone has been shown to directly regulate innate immune cell functions (8), including neutrophil activation and lifespan, highlighting hormonal modulation as a determinant of NETosis propensity in female reproductive tissues.

Neutrophils constitute the predominant leukocyte population of the innate immune system and exert their antimicrobial functions through phagocytosis, degranulation, and the release of neutrophil extracellular traps (NETs) (9). NETs were first described by Brinkmann et al. (10) and represent a distinct antimicrobial strategy characterized by the extrusion of decondensed chromatin decorated with antimicrobial enzymes. The core structural components of NETs include DNA and histones, complexed with granular proteins such as neutrophil elastase (NE) and myeloperoxidase (MPO) (11), forming a meshwork capable of entrapping and neutralizing invading pathogens. In the uniquely dynamic and hormone-responsive environment of the female reproductive tract, NET formation and function may be subject to spatial, temporal, and endocrine regulation, potentially differing from other organ systems. Understanding NET biology in this context is therefore critical for elucidating their roles in gynecologic infections, chronic inflammatory and endocrine-related disorders, and hormone-dependent tumors. These distinct mechanisms provide a basis for how neutrophils and NETs may adapt to the dynamic microenvironments of the female reproductive tract, including cyclical tissue breakdown and repair, as well as hormone-dependent modulation.

In this Review, we summarize NET-mediated immunoregulatory mechanisms in gynecologic diseases, emphasizing their role in specific chronic inflammation and tumorigenesis. We also discuss their potential as diagnostic biomarkers and therapeutic targets within the specialized female reproductive tract microenvironment.

### General NET mechanisms in gynecologic tissues

Building upon the distinctive anatomical, hormonal, and immunological landscape of the female reproductive system discussed above, the biological behavior of neutrophil extracellular traps (NETs) within gynecologic tissues appears to be subject to organ-specific modulation. In contrast to organs with stable structural and hormonal conditions, gynecologic tissues undergo periodic remodeling and endocrine fluctuations, which may impose unique regulatory constraints on NET formation, clearance, and downstream effects. Accordingly, NETs in the female reproductive tract should be interpreted not merely as universal immune effector structures but as context-dependent modulators of tissue immunity and pathology.

### Biological basis of NET formation

**Figure 1:**
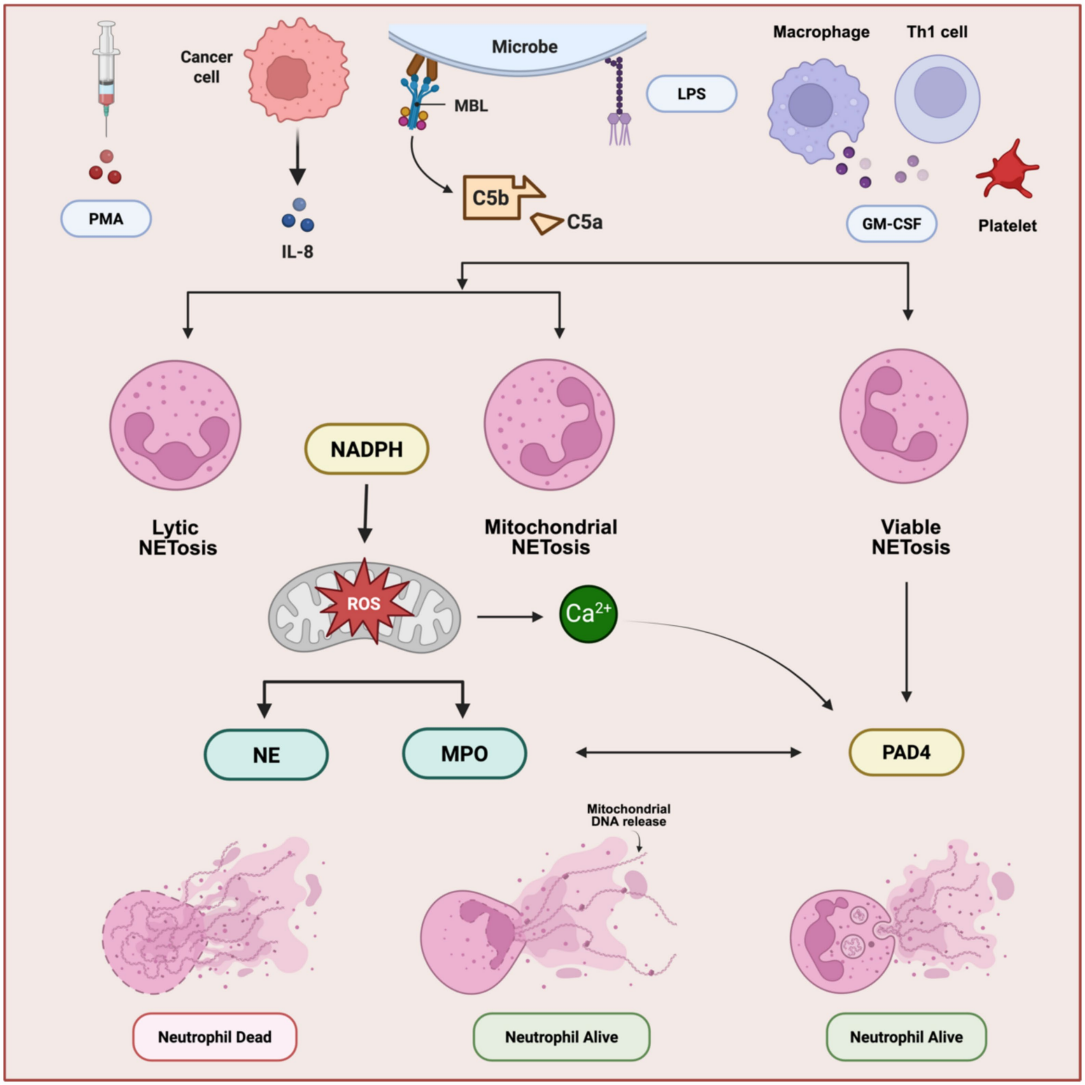
Mechanism of NETs formation. Overview. The illustration depicts three mechanisms of NETs formation: (i) Lytic NETosis, (ii) viable/ROS-independent NETosis, and (iii) mitochondrial NETosis. (i) Lytic NETosis (cell-lytic). Microbial LPS, inflammatory cytokines, or PMA activate surface receptors, triggering the PKC–MAPK signaling pathway. The resulting ROS burst and Ca^2+^ influx activate PAD4, driving histone citrullination and chromatin decondensation. Concurrently, ROS promotes NE and MPO release and nuclear translocation, dismantling the nuclear envelope. Decondensed chromatin bound to granular proteins is expelled through plasma membrane rupture, forming NETs and causing neutrophil death. (ii) Viable NETosis (non-lytic, rapid). Platelet activation, or specific microbes (e.g., *S. aureus*), LPS trigger receptor-mediated signaling or stimulate macrophages and Th1 cells to release GM-CSF, then inducing an early, transient Ca^2+^ spike that rapidly activates PAD4 for limited histone citrullination and selective granule discharge. Unlike lytic NETosis, this pathway is ROS-independent, preserves the nuclear envelope, and exports DNA–protein webs via vesicular exocytosis or membrane blebbing without plasma-membrane rupture. Neutrophils remain viable and retain chemotaxis and phagocytic capacity; NET release occurs within minutes. (iii) Mitochondrial NETosis. In inflammatory settings, immune complexes, or GM-CSF plus C5a promote mitochondrial ROS generation and opening of the permeability transition pore (mPTP) and/or outer-membrane permeabilization (MOMP). Matrix swelling and outer-membrane rupture expel mitochondrial DNA, which complexes with antimicrobial proteins to form extracellular webs. mtDNA can be released before overt cell death or alongside regulated cell-death programs, and is typically more DNase I-sensitive than nuclear DNA-based NETs.

NET formation, termed NETosis, occurs via multiple mechanistic pathways (Figure 1). Classical or lytic NETosis is the best characterized form and is typically induced by strong stimuli such as polymethacrylate (PMA), bacterial pathogens, and immune complexes. This process is dependent on reactive oxygen species (ROS) generation and culminates in nuclear envelope breakdown, chromatin decondensation, plasma membrane rupture, and eventual neutrophil death within approximately 2–4 h (11). In contrast, viable (non-lytic) NETosis is a rapid process occurring within minutes and is largely independent of ROS. In this context, chromatin is released via vesicular transport while preserving plasma membrane integrity, thereby allowing neutrophils to maintain residual effector functions such as chemotaxis and phagocytosis (12). A third mechanism involves the release of mitochondrial DNA rather than nuclear chromatin and has been described particularly in eosinophils and selectively activated neutrophils (13). This pathway is frequently associated with sterile inflammation and non-infectious tissue injury. The coexistence of multiple NETotic pathways provides mechanistic plasticity for neutrophils to adapt to diverse microenvironmental signals, including those uniquely found in the female reproductive tract, such as cyclic hypoxia, hormonal oscillations, fluctuating microbiota, and recurrent tissue remodeling.

### Gynecologic-specific regulation of NET formation

Although NET biology has been extensively studied in systemic immune and inflammatory disorders, emerging evidence suggests that NET formation is highly context-dependent and modulated by the female tissue microenvironment (14). In the female reproductive tract, NET generation is shaped by a combination of anatomical constraints, biochemical conditions, and endocrine cues (14, 15).

Local physicochemical features such as pH gradients, oxygen tension, extracellular matrix (ECM) composition, and mucosal microbiota exhibit pronounced regional variation along the cervicovaginal–uterine axis and influence neutrophil activation thresholds (16). The acidic vaginal milieu, dominated by *Lactobacillus* species, constitutes a barrier distinct from the relatively neutral and dynamic uterine environment, creating site-specific immune niches that differentially regulate neutrophil behavior (16). Perturbation of vaginal microbiota composition has been associated with exaggerated inflammatory responses and enhanced NET release, thereby linking microbial dysbiosis to mucosal immune dysfunction in gynecologic disease (17).

Sex hormones further exert potent regulatory effects on neutrophil recruitment and function. Estrogen and progesterone receptors are expressed on neutrophils and modulate chemotaxis, degranulation, ROS production, and survival (14, 18). Fluctuating estrogen levels across the menstrual cycle modulate neutrophil trafficking and functional polarization, suggesting a role for endocrine signaling in shaping NET dynamics in a cyclical manner (14, 15). Moreover, hormone-dependent pathological states such as endometriosis and gynecologic malignancies generate aberrant endocrine environments that may further reprogram NET responses.

The epithelial barrier states of the cervix and endometrium also impose spatial regulation on NET formation (16). The cyclical disruption and regeneration of the endometrial lining expose stromal and vascular compartments to immune infiltrates, potentially altering NET localization and clearance kinetics (16). In contrast, the cervix operates as a gatekeeper between microbial-rich and normally sterile regions, necessitating tightly controlled neutrophil activation to balance host defense with tissue integrity (18).

Together, these features highlight that NET formation in the female reproductive tract is not merely a passive response to generic inflammatory stimuli, but rather a dynamically regulated process shaped by endocrine cues, microbial ecology, and tissue architecture.

### Dual functional roles of NETs in gynecologic tissues

NETs exert dual and context-dependent functions in immunity, functioning as both innate defense structures and mediators of tissue injury. On one hand, NETs trap and neutralize pathogens, including bacteria, viruses, and fungi, thereby contributing to host defense against infections (10). NET-associated molecules such as DNA and high mobility group box protein 1 (HMGB1) further act as damage-associated molecular patterns (DAMPs), activating dendritic cells and facilitating crosstalk between innate and adaptive immune responses (19, 20).

Conversely, excessive or dysregulated NET formation promotes host tissue injury and amplifies inflammatory cascades. Cytotoxic NET components, including NE, MPO, histones, and extracellular DNA, compromise epithelial integrity, trigger oxidative stress, and sustain inflammatory signaling (21). NETs also expose nuclear and post-translationally modified auto-antigens, including citrullinated proteins and double-stranded DNA, thereby contributing to immune tolerance breakdown and autoantibody production, as observed in autoimmune conditions such as systemic lupus erythematosus (SLE) (22, 23). Moreover, NETs promote complement activation and coagulation cascades, facilitating immunothrombosis and chronic inflammatory pathology in multiple disease contexts (11, 24).

In gynecologic tissues, the balance between protective and pathogenic NET functions is uniquely shaped by cyclical physiological injury, endocrine modulation, and dynamic tissue remodeling. In infectious conditions, NETs restrict microbial dissemination within the cervicovaginal or uterine mucosa; however, excessive NET release may impair epithelial regeneration and compromise fertility by inducing sustained mucosal damage (25). Under sterile inflammatory conditions, aberrant NET formation contributes to a pro-inflammatory microenvironment associated with chronic pelvic pain and fibrotic remodeling, although causal relationships remain incompletely defined (26).

Tumor-associated inflammation also drives NET formation and persistence. Tumor-derived factors induce neutrophil activation and NETosis, fostering peritumoral niches that facilitate cancer progression. NET-mediated signaling through pathways such as TLR/NF-κB promotes cytokine release, epithelial–mesenchymal transition (EMT), endothelial dysfunction, and vascular remodeling, thereby enhancing tumor invasion, metastasis, and immune evasion (11).

Notably, many gynecologic malignancies exhibit strong hormone dependence, indicating that endocrine, immune interactions may uniquely regulate NET dynamics in female-specific tumors. Estrogen- and progesterone-enriched tumor microenvironments may reshape chromatin accessibility, inflammatory signaling, and cellular metabolism, implicating NETs as critical nodes within an emerging hormone–immune–chromatin crosstalk network. These findings support the concept that NETs in gynecologic cancers operate within regulatory circuits that differ fundamentally from those observed in non-hormone-responsive malignancies.

## NETs in infectious and sterile inflammatory diseases

**Figure 2:**
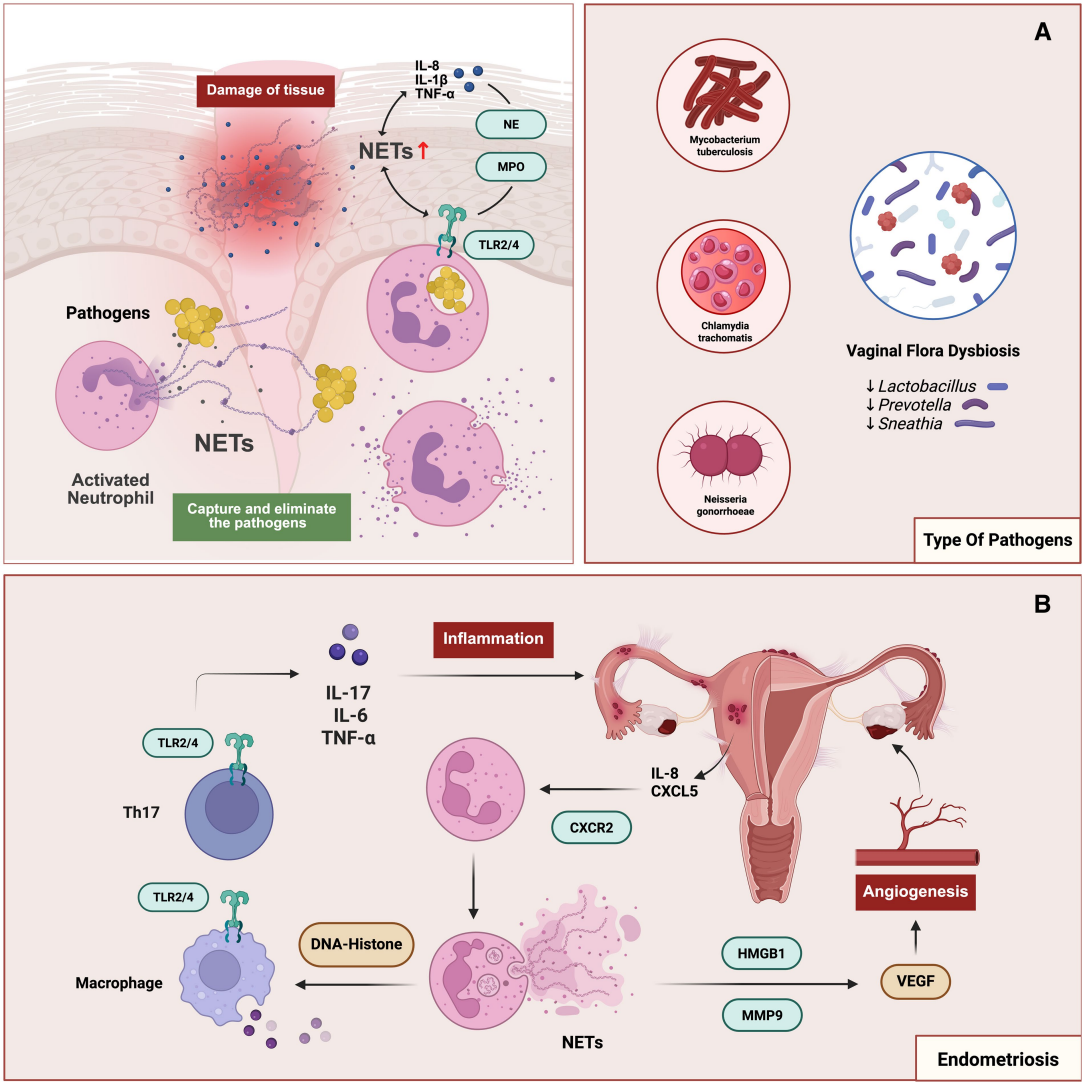
NETs in infectious and sterile inflammatory diseases. **(A)** Pathogenic microorganisms (e.g., *Mycobacterium tuberculosis*, *Chlamydia trachomatis*, *Neisseria gonorrhoeae*) and vaginal microbiota dysbiosis (reduced *Lactobacillus*, increased *Prevotella* and *Sneathia*) activate neutrophils via TLR2/4 signaling, leading to NETs release enriched with NE and MPO. NETs contribute to pathogen capture and clearance but also amplify inflammatory responses through the induction of IL-8, IL-1β, and TNF-α. **(B)** In endometriosis, NET-associated components (DNA–histone complexes, HMGB1, MMP9) and pro-inflammatory cytokines (IL-17, IL-6, TNF-α) derived from Th17 cells and macrophages promote chemotaxis of neutrophils (via IL-8/CXCL5–CXCR2 axis), sustaining inflammation. Concurrently, NETs facilitate angiogenesis by upregulating VEGF, thereby supporting ectopic endometrial lesion establishment and progression.

Inflammatory gynecological diseases arise from both infectious and sterile origins and represent a major cause of pelvic pain, infertility, and reproductive organ dysfunction. While infectious disorders such as pelvic inflammatory disease (PID), genital tuberculosis (GTB), and vaginitis are typically driven by microbial pathogens, sterile inflammation, exemplified by endometriosis or tissue injury, reflects dysregulated immune activation independent of overt infection (Figure 2). Despite distinct etiologies, accumulating evidence indicates that these conditions converge on aberrant NET formation as a shared pathogenic effector, although disease-specific triggers and downstream consequences differ substantially.

### NETs in infectious gynecological inflammation

PID is a polymicrobial inflammatory disorder of the female upper genital tract, most commonly initiated by ascending infection with sexually transmitted pathogens, including *Neisseria gonorrhoeae* and *Chlamydia trachomatis.* Clinically, it presents with pelvic pain, cervical motion tenderness, and abnormal discharge, often complicated by endometritis and salpingitis (27). Although direct evidence in human PID remains scarce, animal models in cows and mares consistently demonstrate robust NET formation at the site of genital infection (28, 29). This suggests an evolutionary conserved antimicrobial response in the female reproductive tract.

At the molecular level, pathogen-activated neutrophils undergo ROS-dependent lytic NETosis, mitiated by Protein Kinase C (PKC) and calcium signaling-mediated activation of NADPH oxidase, which promotes chromatin decondensation and extracellular extrusion (30). This response is further amplified by pro-inflammatory cytokines such as IL-8, IL-1β, and TNF-α (31). While NETs entrap bacteria, their enzymes increase vascular permeability, induce endothelial injury, edema, and fibrin deposition, promoting adhesion formation and tubal scarring (31). Moreover, NET components engage TLR2/4 on macrophages and T helper cells, sustaining IL-6, TNF-α, and IL-17 production (32, 33). This sustained NET–immune feedback loop likely underlies the transition from acute infection to chronic pelvic inflammation and promotes sequelae such as peri-tubal adhesions and tubo-ovarian abscesses such as peri-tubal adhesions and tubo-ovarian abscess formation (31).

Genital tuberculosis represents another paradigm in which pathogenic NETosis contributes to reproductive dysfunction. *Mycobacterium tuberculosis* (Mtb) typically disseminates from pulmonary sites (34), induces ROS-dependent NETosis through PKC/calcium signaling, while PAD4-mediated histone citrullination facilitates chromatin extrusion (35). Type I interferons further facilitate this process (36). Although NETs immobilize Mtb within DNA scaffolds, paradoxically, NETosis may aid bacterial survival through inhibition of PAD4, or pharmacologic disruption of NETs reduces Mtb load in vitro (36, 37). Additionally, Mtb-induced NETs stimulate macrophages via TLR2/4, promoting IL-1β, IL-6, TNF-α and IL-10 production and intense inflammation (35). Experimental and clinical evidence indicate that dysregulated NETosis in GTB is associated with mucosal destruction, stromal fibrosis, and fallopian tube obstruction, providing a pathological basis for infertility and menstrual dysfunction in tuberculosis (35–37). Thus, although NETs may initially constrain Mtb dissemination, their chronic activation appears to drive fibrotic remodeling. Consequently, anti-NETs drugs represent a promising host-directed therapeutic strategy for GTB in the future.

Vaginal dysbiosis further exemplifies how microbial imbalance fuels pathological NET formation. Vaginal samples from women with *Vulvovaginal candidiasis*, *Candida albicans*, and *Trichomonas vaginalis* showed marked NET accumulation, with trichomoniasis exhibiting the highest NET density (25). Likewise, enhanced NETosis was observed in intermediate microbiota states and bacterial vaginosis (BV), with NETs markers significantly elevated in BV patients but nearly absent in healthy women (25). These findings suggest that loss of L*actobacilli* dominance and overgrowth of anaerobes species such as *Gardnerella* and *Atopobium* is strongly associated with increased NETosis (38).

Mechanistically, cervical mucus enriched in sialylated mucins normally suppresses NETosis via Siglec–glycan interactions. BV-associated bacterial degrade this protective barrier, thereby promoting NET release (39). In women with polycystic ovary syndrome (PCOS) and concurrent vaginal dysbiosis, NETs markers are further elevated, linking endocrine–microbiota interactions to innate immune dysregulation (26). Although NETs entrap and kill vaginal pathogens, excessive NETosis drives epithelial injury by activating TLR2/4 on macrophages and Th17 cells, triggering IL-6 and TNF-α release, and perpetuating mucosal inflammation (32, 40). In summary, microbial dysbiosis removes local immune restraints, exposing vaginal mucosa to NET-driven inflammatory damage.

### NETs in gynecological sterile inflammation

Endometriosis is a chronic, estrogen-dependent inflammatory disorder characterized by ectopic implantation of endometrial tissue and manifests clinically as pelvic pain and infertility (41). Ectopic endometrial lesions are heavily infiltrated by activated neutrophils and NETs, intensifying the local inflammatory microenvironment (26). NET-derived histones, MPO, and NE activate signaling that promotes lesion cells in proliferation and angiogenesis (42). while circulating and local NET markers, particularly citH3, are elevated in plasma and peritoneal fluid of endometriosis patients, especially those with deep infiltrating disease (26, 43, 44). Inflammatory cytokines produced by lesions, including IL-8 and CXCL5, recruit neutrophils through CXCR2 and trigger NETosis. Pharmacologic blockade of the CXCL5-CXCR2 axis markedly reduces NETs density and lesion adhesion in animal models (43). Beyond inflammatory amplification, NETs function structurally as an adhesive scaffold for ectopic cells, facilitating endometrial cell attachment (45) promote angiogenesis via MMP9 and HMGB1 mediated VEGF signaling (46). Meanwhile, NET-DNA-histone complexes stimulate TLR2/4 on macrophages and Th17 cells, reinforcing IL-6, IL-17, and TNF-α release and thus sustaining a self-propagating inflammation circuit (32).

Recent studies also reveal a critical interplay between NETs and the NLRP3 inflammasome (47). NLRP3, which consists of ASC and pro-caspase-1, forms a key inflammatory platform in mast cells, macrophages, and endothelium (48). ROS and NF-κB upregulate NLRP3, activating caspase-1 and releasing IL-1β and IL-18, along with gasdermin D-dependent pyroptosis, and fueling inflammatory DAMP release and active NETosis (49). Notably, estrogen signaling through ER-α enhances NLRP3 activation and IL-1β production in mast cells, thus promoting angiogenesis and fibrosis, whereas progesterone suppresses NLRP3 via autophagy. However, ectopic endometrial cells often resist this regulation (50). Inhibiting NLRP3 with MCC950 can reduce lesion size and cytokine release in murine models, supporting a feed-forward interaction between inflammasome activation and NETosis (49). These data suggest that NET formation and NLRP3 activation reinforce each other. This complex network (involving CXCR2–CXCL5, PAD4, TLR– NF-κB, NLRP3, integrins/MMP9, estrogen signaling) emerges that sustains lesion growth, angiogenesis, fibrosis, and possibly EMT in endometriosis.

Microbial factors may further modulate this axis. Fusobacterium infection has been shown to accelerate endometriosis progression by inducing fibroblast phenotypic transition through the NET- and TGF-β signaling pathway (51), implicating dysbiosis as a co-driver of sterile inflammation.

## NETs in reproductive endocrine-related diseases

**Figure 3:**
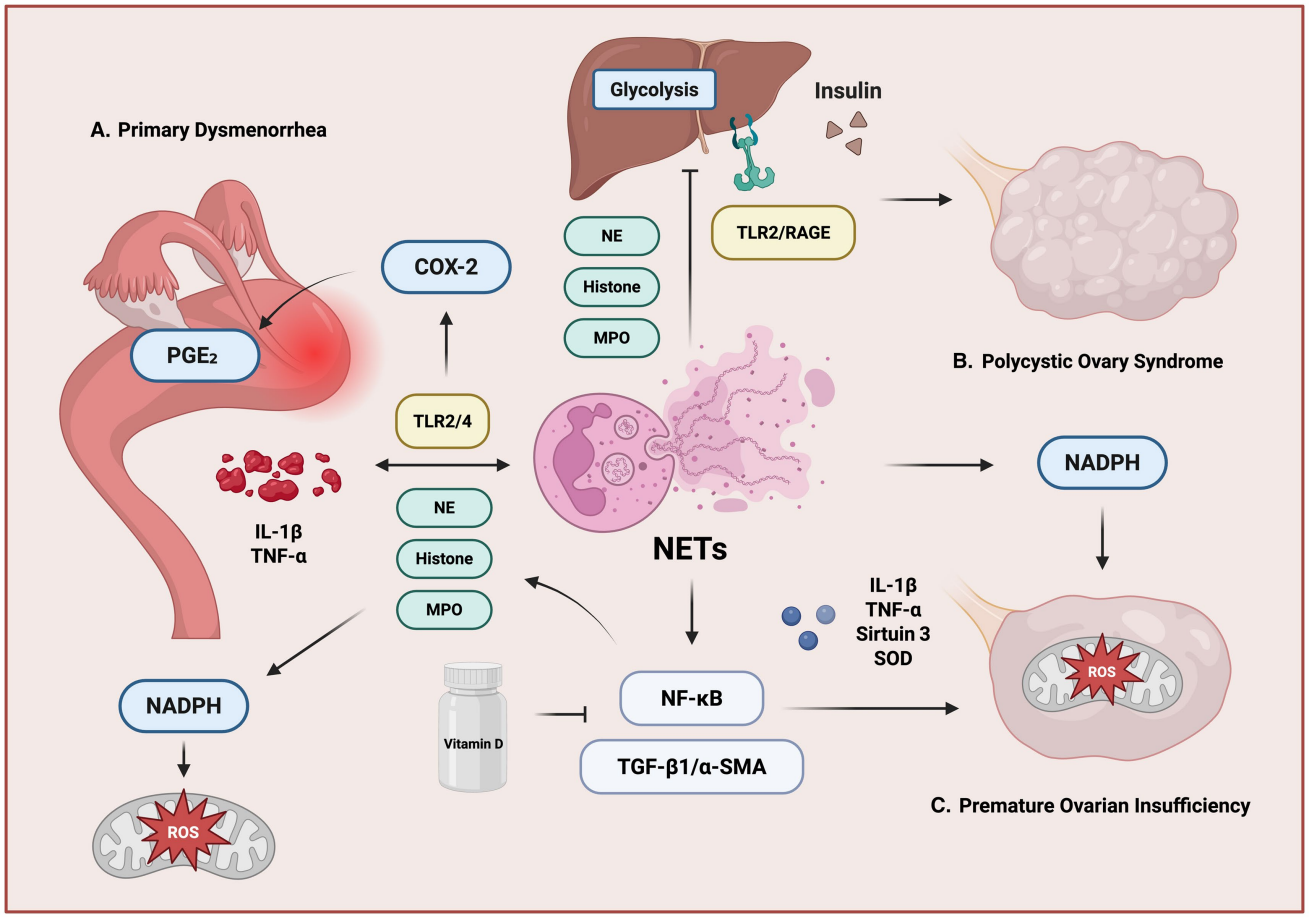
NETs in reproductive endocrine-related diseases. **(A)** In PD, inflammatory mediators (IL-1β, TNF-α) stimulate PGE₂ production via COX-2 activation through TLR2/4 signaling. PGE₂ and cytokine-induced NADPH oxidase activity increase ROS, promoting NET release enriched with NE, histones, and MPO. **(B)** In PCOS, insulin resistance and altered glycolysis engage TLR2/RAGE pathways, enhancing NET formation and systemic inflammation. **(C)** In POI, NET-associated pro-inflammatory cytokines (IL-1β, TNF-α) and ROS impair ovarian function, potentially mediated by Sirtuin 3 downregulation and reduced SOD activity. NET-driven activation of NF-κB and TGF-β1/α-SMA signaling may contribute to ovarian fibrosis and endocrine dysfunction. Vitamin D supplementation may attenuate these effects by modulating NET formation and inflammatory pathways.

Reproductive endocrine-related diseases encompass a spectrum of gynecological conditions primarily driven by hormonal imbalances, including PCOS, premature ovarian insufficiency (POI), and primary dysmenorrhea (PD). These disorders represent major causes of female infertility and are frequently associated with ovulatory dysfunction, aberrant steroidogenesis, and chronic inflammation. While traditionally considered as endocrine or metabolic syndromes, accumulating evidence reveals a strong interplay between hormonal imbalance and immune dysregulation (Figure 3).

PCOS is a common endocrine disorder of reproductive-age women, and id defined by anovulation, hyperandrogenism, and polycystic ovarian morphology (52). PCOS is increasingly recognized as a chronic low-grade inflammation condition. Elevated NETosis has been documented systemically, and within the ovarian microenvironment of affected women, and circulating as well as follicular NET markers are significantly increased in PCOS patients (53, 54).

Metabolic dysregulation in PCOS is typified by insulin resistance, which elevates the morbidity of type 2 diabetes (T2DM) (55). Indeed, over 50% of PCOS patients develop T2DM by age 40 (55). NETs disrupt hepatic insulin signaling by downregulating key glycolytic enzymes, reducing glucose uptake and utilization (53). In a DHEA-induced PCOS rat model, DNase I markedly improved insulin sensitivity and glucose tolerance (53). NETs also harm ovarian function by inducing apoptosis in ovarian granulosa cells, compromising oocyte quality, and further impairing the ovarian milieu through sustained inflammatory signaling, exacerbating PCOS pathophysiology (53).

POI is defined by early-onset loss of ovarian function before the age of 40, manifesting as amenorrhea, hypoestrogenism, and elevated gonadotropins (54). Although the etiology of POI is heterogeneous, growing evidence suggests that NETs participate in ovarian failure through coordinated inflammatory, oxidative, and fibrotic mechanisms. NET-associated oxidative stress promotes apoptosis of granulosa and contributes to follicular atresia (56). In parallel, NET-related inflammatory signaling sustains cytokine production while suppressing intracellular antioxidant defense, thereby amplifying cellular damage (57). NETosis also upregulates TGF-β1/α-SMA signaling, leading to ovarian fibrosis (58). These NET-mediated processes collectively deplete follicles and impair ovarian function in POI.

Interestingly, vitamin D has been shown to attenuate NET formation while simultaneously suppressing inflammatory and profibrotic signaling and boosting antioxidant defenses (58, 59). In animal models, Vitamin supplementation can reduce NETs density, lead to a decrease in ovarian fibrosis, and preserve follicle counts (59). Notably, a traditional Chinese medicine called *Danggui Buxue Tang* exerts similar protective effects in ovarian failure by raising Vitamin D levels and inhibiting NETosis (60).

PD is characterized by excessive endometrial prostaglandin production, most notably prostaglandin E₂ (PGE₂), leading to uterine hypercontractility, ischemia, and menstrual pain (61). This condition is associated with systemic oxidative stress, evidenced by elevated serum malondialdehyde levels and reduced antioxidant defenses. Although direct mechanistic studies remain limited, accumulating data suggest that NET-associated inflammatory and oxidative pathways amplify uterine nociception. Components of NETs, especially MPO, NE, histones, and DNA, promote oxidative stress and inflammatory signaling within the endometrial milieu and thereby exacerbate dysmenorrhea (21). During menstruation, high local concentrations of IL-8 and TNF-α create a permissive environment for NET induction, reinforcing inflammatory signaling loops (21).

Furthermore, NET-derived histones have been implicated in the upregulation of cyclooxygenase enzyme-2 (COX-2) via TLR activation (62), then enhancing PGE₂ synthesis and activating the nociceptive neurons (63) (sensory neurons which can sense and transmit pain signals), ultimately increasing uterine contraction frequency and pain intensity. However, pharmacological compounds with PGE2 can also inhibit NETosis through the cAMP-PKA pathway and in a protein kinase A-dependent manner (63).

## NETs in gynecologic tumors

Gynecologic tumors comprise a heterogeneous group of malignancies arising from the female reproductive tract, including cervical cancer, endometrial cancer, ovarian cancer, and gestational trophoblastic neoplasia. While these tumors differ in histogenesis and clinical behavior, they share common hallmarks of cancer such as chronic inflammation, angiogenesis, immune dysregulation, and extracellular matrix (ECM) remodeling. Recent studies have uncovered a pivotal role of neutrophils and NETs in shaping the tumor microenvironment.

There are several shared common NET-mediated mechanisms in gynecological tumors. NETs influence tumor biology through a broadly conserved cascade that parallels mechanisms described in other solid tumors, infections, and chronic inflammatory diseases, yet operates within an endocrine-responsive reproductive context. Firstly, tumor and host-derived stimuli, including IL-8, G-CSF, IL-1β, TNF-α, microbial products, and damage-associated molecular patterns, which prime tumor-associated neutrophils and induce ROS- and PAD4-dependent NETosis (64–68). Secondly, the DNA-histone backbone of NETs forms a physical scaffold that traps circulating or free-floating tumor cells via β1- and αv-integrins, promotes their adhesion to endothelial or mesothelial surfaces, and thereby seeds metastatic niches in lymph nodes, the peritoneal cavity, and distant organs (68–70). This adhesive function is particularly relevant for lymphatic spread in cervical cancer and transcoelomic dissemination in ovarian cancer. Thirdly, NET-associated proteases (NE, MMP-9) and other enzymes remodel the ECM, expose cryptic adhesive motifs, and activate EMT and survival-related signaling pathways such as PI3K/AKT and FAK/ERK/YAP, thereby promoting invasion, angiogenesis, and treatment resistance (71, 72).

Furthermore, NETs reshape the immune tumor microenvironment (TME). By forming aggregates with circulating tumor cells and platelets, they sterically shield cancer cells from immune recognition, while NET-derived histones and proteases directly impair CD8^+^ T-cell and NK-cell cytotoxicity and foster up-regulation of immune checkpoints, including PD-L1 on tumor or myeloid cells (73). These effects collectively support immune escape and may attenuate responses to immune checkpoint blockade. Additionally, excessive NETosis spills into the circulation. Systemic markers such as cfDNA/dsDNA, citH3, NE, and MPO, together with hematologic indices like the neutrophil-to-lymphocyte ratio (NLR), correlate with tumor burden, advanced stage, therapy resistance, and poor survival across cervical, ovarian, and endometrial cancers (74–79). In endometrial cancer, NET-derived cfDNA/dsDNA and characteristic long DNA fragments provide additional prognostic granularity beyond classical clinicopathologic factors (71, 80).

**Figure 4:**
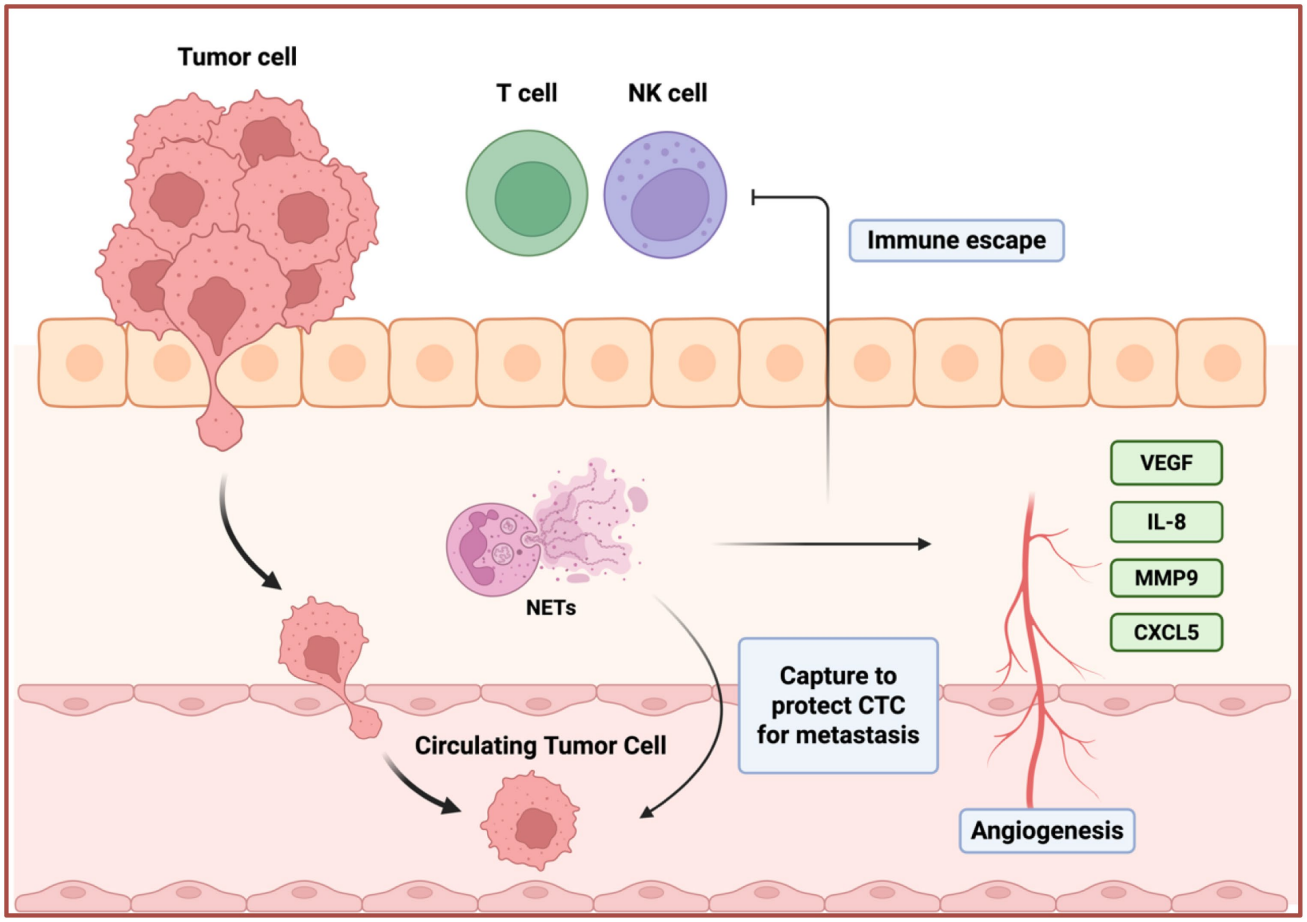
NETs in gynecological cancer progression and metastasis. NETs promote metastasis in gynecological cancers through circulating tumor cells capture, immune evasion, and angiogenesis. NETs physically capture CTCs, shielding them from immune surveillance and promoting their survival and extravasation. Concurrently, NETs contribute to immune evasion by impairing the cytotoxic functions of T cells and NK cells. The tumor microenvironment further supports metastasis through the secretion of pro-angiogenic factors, including VEGF, IL-8, MMP9, and CXCL5, which drive angiogenesis to establish a nutrient-rich niche for tumor growth and dissemination.

**Figure 5:**
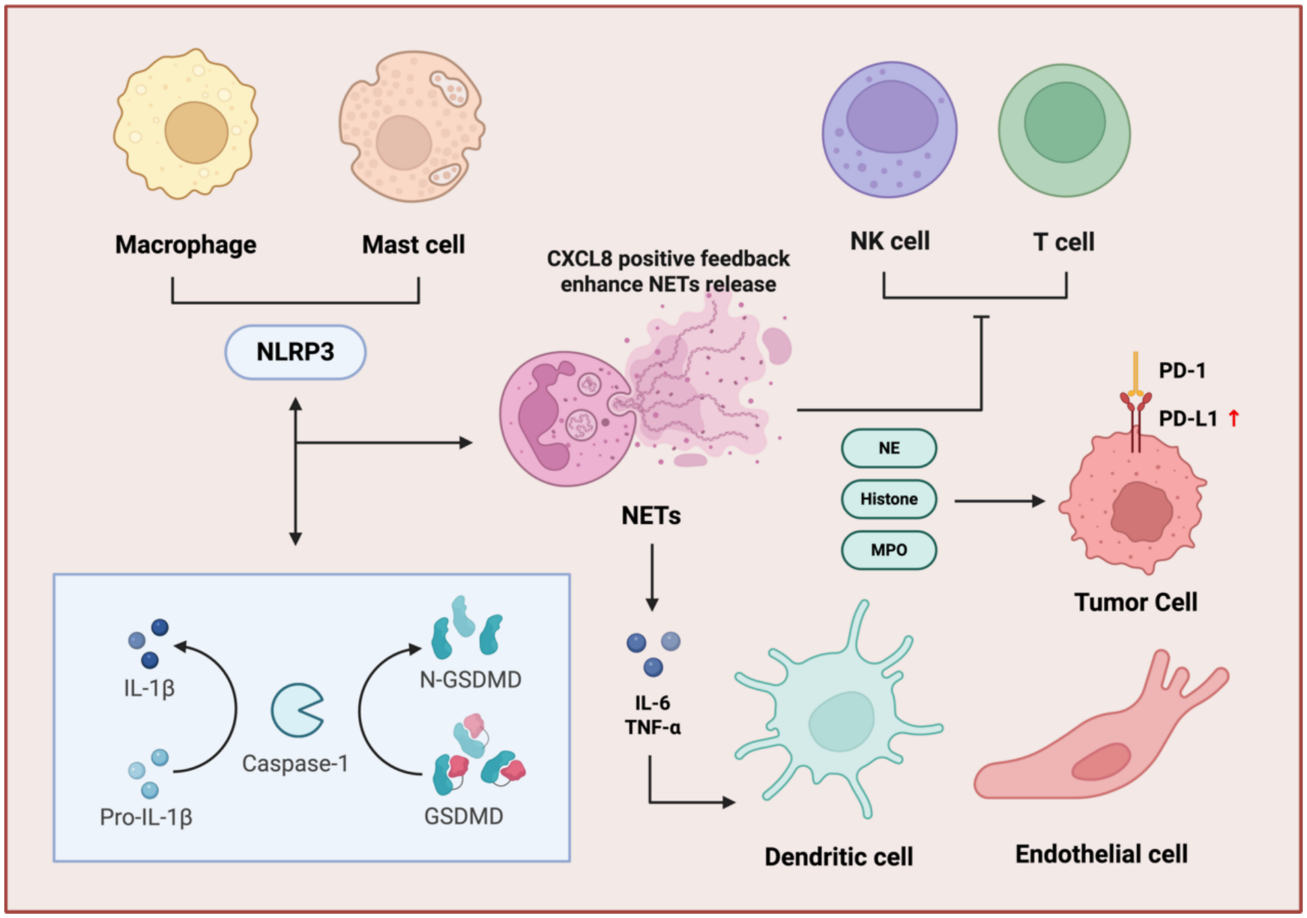
Immune cell regulatory network of NETs. NETs amplify immunosuppression through inflammasome activation, cytokine feedback, and immune checkpoint dysregulation. NET components, NE, histone, and MPO stimulate macrophages and mast cells to activate the NLRP3 inflammasome, triggering GSDMD into N-GSDMD and maturation of IL-1β. This cascade enhances NET release via a CXCL8-positive feedback loop. Concurrently, NETs induce pro-inflammatory cytokines (IL-6, TNF-α) and upregulate PD-L1 on tumor cells, promoting T cell and NK cell exhaustion through PD-1/PD-L1 interactions. Dendritic and endothelial cells further contribute to this immunosuppressive microenvironment, facilitating tumor immune evasion.

Crucially, these shared NET-driven processes occur in reproductive organs that are continuously exposed to fluctuating levels of estrogen and progesterone, cervicovaginal microbiota, ovulatory injury, and pregnancy-like immunologic states (64). Experimental studies indicate that estradiol can lower the threshold for NET formation by enhancing neutrophil activation and PAD4-dependent chromatin decondensation (72), whereas progesterone antagonizes this pro-NETotic effect in pregnancy models and viral airway inflammation (81). Together with data from endometriosis and pregnancy complications, showing NET enrichment in hormonally active environments (45). These findings support the concept that NET programs in gynecologic tumors are not simply generic inflammatory responses, but are modulated by sex hormones and reproductive states in ways that distinguish them from NET biology in non-gynecologic solid malignancies (Figures 4, 5).

In cervical cancer (CC), NETs influence tumor growth and patient outcomes. Issue studies show that MPO and citH3 are significantly increased in tumor specimens, and these NET components are elevated in the peripheral blood of CC patients compared with healthy controls (65). Yan et.al demonstrated that high stromal density of NETs independently predicts worse recurrence-free survival (RFS) (74), indicating that NETs are not merely a byproduct of inflammation but an active prognostic determinant.

Cervical tumor cells actively induce NETosis and promote invasion and metastasis. For instance, S100A7-overexpressing CC cells can recruit neutrophils and trigger ROS-dependent lytic NETosis (66). These NETs then engage TLR2 and activate p38-MAPK/ERK /NF-κB signaling in tumor cells, enhancing motility, increasing lymphatic permeability, and promoting lymph angiogenesis, thereby facilitating lymph node metastasis. This process can be attenuated by DNase I or by inhibiting TLR2 with chloroquine (66). Tumor-derived IL-8 similarly recruits neutrophils via CXCR1/2 and induces NETs that promote angiogenesis, immune evasion, and micrometastatic niche formation (67). Also, the DNA backbone of NETs, via integrin-binding domains (α5β1, αvβ3), captures circulating tumor cells, while MMP-9 and NE remodel the ECM and activate FAK/ERK/YAP signaling, amplifying metastatic potential (70).

Persistent high-risk HPV infection and chronic inflammation are central drivers of cervical carcinogenesis. Vaginal dysbiosis produces increased short-chain fatty acids (SCFAs), which activate NF-κB, raise ROS levels, and stimulate the release of pro-inflammatory cytokines (75), thereby intensifying local inflammation, disrupting the epithelial barrier, and promoting HPV persistence and progression of cervical intraepithelial neoplasia (CIN) (76). Persistent HPV infection further disrupts local immune homeostasis, which means the E6/E7 oncoproteins impair NF-κB and STAT3 pathways, suppress interferon signaling via TLR9 modulation, and induce epigenetic silencing of immune mediators, reinforcing mutual reinforcement between microbial dysbiosis and HPV pathogenicity (77–79). At the mucosal level, IL-8 potently induces NETosis, NET components inflict epithelial injury and generate DNA double-strand breaks, thereby facilitating HPV genome integration by engaging the ATM/ATR-mediated DNA damage response (DDR) (82). Continuous expression of E6/E7 further impairs p53/Rb regulation while sustaining NF-κB/STAT3 activation, thus maintaining an immunosuppressive, pro-inflammatory microenvironment (83). This sequence establishes a self-reinforcing loop: vaginal dysbiosis → chronic inflammation → NETosis → epithelial damage and DDR activation → HPV integration and oncogene expression → increased inflammation and barrier disruption → further dysbiosis and NETosis, collectively driving progression from CIN to invasive carcinoma. Although direct studies prove that NET-mediated DNA damage promotes HPV integration in CC is still lacking, evidence from other cancers strongly supports NET-induced DNA damage, DDR activation, and chromatin remodeling, making this axis biologically plausible (82).

Clinically, NLR serves as an independent prognostic marker in cervical cancer, correlating with poorer overall and progression-free survival (PFS), higher tumor grade, advanced stage, vascular invasion, lymph node metastasis, and therapy resistance, a consistent finding across multiple cohorts (84). Tumor-associated neutrophils promote NETosis via TLR4 and IL-8/CXCR1/2 pathways and suppress CD8^+^ T-cell infiltration (73) while NET-associated DNA damage response may contribute to chemoradiotherapy resistance (85).

In ovarian cancer (OC), especially high-grade serous ovarian carcinoma (HGSOC), NETs appear to be particularly important in the peritoneal and omental dissemination that characterizes this disease. OC cells secrete IL-8 and G-CSF to induce PAD4-dependent NETosis and extrusion of citH3-rich webs (68). These NETs efficiently capture free-floating OC cells in the peritoneal cavity and omentum, then facilitate their stable adhesion to mesothelial surfaces (68). Lee observed abundant NETs in omental tissue from both mouse models and early-stage OC patients, and blockade of NET formation with PAD4 inhibitors or DNase I significantly reduces omental metastatic (69). Patients with G-CSF-driven neutrophilia exhibit particularly extensive peritoneal NETs and advanced dissemination, linking a specific cytokine axis to NET-mediated spread (68). Furthermore, NET-associated enzymes have been proven in OC to be capable of activating EMT and PI3K/AKT signaling to promote the proliferation and invasion (11, 86).

Beyond promoting physical dissemination, NETs also orchestrate an immunosuppressive milieu. They can form aggregates with circulating tumor cells (CTCs), often in concert with platelets, which physically shield cancer cells from immune surveillance and clearance (62, 87). Moreover, NET-derived histones and proteases can directly impair the cytotoxic functions of T cells and natural killer (NK) cells, in part by up-regulating the expression of immune checkpoint proteins like PD-L1 on tumor cells (73, 88).

Given their pro-tumorigenic activities, NETs have been rigorously investigated as potential biomarkers for OC. For instance, Singel and his team reported that elevated levels of mitochondrial DNA (mtDNA) and NE in the ascites of patients with HGSOC correlated with significantly shorter PFS (89). Sarai and her team’s recent work corroborates these findings, showing that HGSOC patients presented a higher concentration of cfDNA, citH3, and calprotectin in plasma and of all five NETosis biomarkers in peritoneal fluid (PF) than control women (90). Moreover, these biomarkers showed a strong ability to differentiate the two clinical groups. Interestingly, they also found that neoadjuvant treatment seemed to reduce NETosis biomarkers mainly in plasma compared to PF (90). However, the prognostic implications are not always straightforward. A multi-omics analysis by Muqaku revealed that HGSOC ascites with high NET marker concentrations were linked to a robust local inflammatory response and better patient survival. The diagnostic potential of circulating NET components also remains uncertain, as Dobilas found that plasma levels of H3Cit or dsDNA did not offer superior performance over the conventional marker CA125 for early OC detection (87).

Moving from fluid biomarkers to genomics, NET-related gene expression signatures have emerged as powerful prognostic tools. Researchers have developed a prognostic marker of eight genes (including ELN, FBN1, IL1B, LCN2, MMP2, MMP9, RAC2, and SELL) (91). It has been confirmed that the NETs-related prognostic marker is a reliable tool for predicting drug sensitivity and evaluating immune characteristics in ovarian cancer patients, thereby facilitating decision-making in ovarian cancer management. Notably, they have identified the significance of RAC2, one of the NETs-related marker genes, in ovarian cancer metastasis, which may provide clues for the anti-tumor response of NETs (91). Similarly, a prognostic model based on NET-associated long non-coding RNAs (lncRNAs) accurately predicted patient responses to combination therapies involving PARP inhibitors and PD-1/PD-L1 blockade (92). These genomic models underscore that heightened NET activity is indicative of an immunosuppressive TME and predicts resistance to immunotherapy, bolstering the case for using these signatures to complement existing stratification methods like PD-L1 expression, BRCA ½, and homologous recombination deficiency (HRD) status (93).

The multifaceted roles of NETs in promoting OC progression make them a compelling and druggable therapeutic axis. Pre-clinical studies have consistently demonstrated the efficacy of targeting NETs directly. As shown by Lee, inhibiting NET formation with PAD4 inhibitors or degrading their DNA backbone with DNase I significantly curtails omental metastasis (94). Moreover, targeting NETs holds promise for overcoming therapeutic resistance. NETs have been implicated in the development of chemoresistance by acting as reservoirs for latent TGF-β, which is subsequently activated by MMP-9 to initiate EMT. Targeting NETs has been shown to disrupt this axis, thereby re-sensitizing tumor cells to chemotherapy (95). In the context of immunotherapy, systemic administration of DNase I can enhance CD8 + T-cell infiltration and reverse resistance to PD-1 blockade, partly by restoring the function of exhausted T cells (96, 97). Consequently, therapeutic strategies are expanding beyond direct NET disruption to include targeting upstream signaling pathways (the IL-1β or IL-8/CXCR2 axes that trigger NETosis) or downstream angiogenesis and metastasis, which is promoted via the CCDC25 receptor on OC cells (86).

While most evidence remains pre-clinical, these findings provide a strong rationale for clinical translation. For instance, recombinant human DNase I, an approved therapy for cystic fibrosis, is being explored in oncology trials for other solid tumors, and novel PAD4 inhibitors are advancing through early-phase clinical development (98). The safety and efficacy of these NET-targeting agents in the OC setting, particularly in combination with standard-of-care treatments like PARP inhibitors and ICIs, represent a critical and promising area for future investigation (99). Combining NET inhibition with anti-angiogenic agents like bevacizumab could also yield synergistic effects, further highlighting the potential of this strategy.

Endometrial cancer (EC) typically arises in the background of chronic inflammation and metabolic-endocrine dysregulation, particularly obesity, insulin resistance, and unopposed estrogen exposure (64). These conditions provide a fertile microenvironment for NET induction within the endometrium. Ronchetti demonstrated pervasive NETosis across all EC histological grades (G1–G3), with citH3, NE, and histone H2B labeling tumor-infiltrating neutrophils and extracellular fibrillary networks (80). Quantitative analysis revealed that the proportion of citH3-positive leukocytes increases with tumor grade, whereas normal endometrium showed no NET formation, indicating that NETosis intensity tracks with tumor aggressiveness (80).

A distinctive feature of EC compared with other gynecologic tumors is the rich information carried by circulating NET-related DNA markers. Seo measured three plasma NET markers – histone-DNA complexes, cell-free DNA (cfDNA/dsDNA), and NE—in 98 EC patients and 45 healthy controls (71). They found that all three markers were significantly higher in EC patients than in healthy controls, with diagnostic performance comparable to serum CA125 (71). Ronchetti also reported that EC patients had increased total cfDNA and decreased cell-free mitochondrial DNA (cf-mtDNA), and that the combination of these measures could effectively differentiate patients from controls (80). Notably, Seo was the first to demonstrate the independent prognostic value of cfDNA/dsDNA in EC (71). Univariate analysis showed that high cfDNA/dsDNA levels were associated with shorter PFS and overall survival (OS) after adjusting for FIGO stage, histological type, lymphovascular space invasion, and lymph node status, establishing cfDNA/dsDNA as an adverse prognostic factor (71).

In contrast, Ronchetti found no significant differences in serum citH3 across patient grades or compared to controls—a discrepancy likely reflecting divergent dynamics of local NETosis versus systemic biomarker levels, as well as differential clearance mechanisms (80). Notably, they observed positive correlations between serum citH3 and cfDNA, and negative correlations with cf-mtDNA (most pronounced in G1/G2 stage) (80). Microfluidic fragment analysis further revealed that a subset of EC patients exhibited long cfDNA fragment peaks (~1,000–9,050 bp), such cfTNP-positive samples were associated with higher citH3 and total cfDNA but lower cf-mtDNA levels (80). Moreover, cfTNP presence correlated with elevated lymphocyte counts and fibrinogen levels, especially in low to intermediate grade EC (80). These data suggest that long cfDNA fragments may partly originate from NETosis (particularly in low-to intermediate-grade EC) and may reflect impaired NET clearance. The fragment size pattern, in combination with citH3 levels, could thus help distinguish low-grade (G1/G2) from high-grade (G3) EC.

Ronchetti further reported correlations between NET markers and systemic inflammatory/hematological parameters: serum citH3 correlated positively with lymphocyte counts, and cfDNA levels correlated positively with monocyte counts, lymphocyte counts, platelet counts, and fibrinogen levels (80). In analyses stratified by grade, cfDNA levels in G1 patients correlated with neutrophil counts and fibrinogen. In G2 patients, cfDNA correlated with lymphocyte counts and fibrinogen, and EC patients with the cfTNP pattern tended to have higher lymphocyte counts and fibrinogen levels (80). Given the robust epidemiological and mechanistic links between obesity and EC, which are mediated through chronic, low-grade inflammation in white adipose tissue, researchers have increasingly investigated whether adiposity-driven inflammatory signals promote NET formation within the EC microenvironment (64). Seo pointed out that, although they did not directly correlate systemic inflammation with NET biomarkers, obesity-related inflammation and adipokines likely drive NETosis in EC (71), citing mechanistic support from Moukarzel, who demonstrated that obesity-induced adipose inflammation elevates CRP, IL-6, and TNF-α, thus stimulating NETs release (64). Furthermore, Abakumova showed that neutrophils from early-stage (FIGO IA) EC patients have an increased predisposition to produce NETs but a diminished tumor-cell trapping ability, suggesting dysfunctional NETs may facilitate metastatic spread (100).

In gestational trophoblastic neoplasia (GTN), such as choriocarcinoma, remains limited, but the disease develops in a pregnancy-like immune and hormonal context that has been extensively studied in obstetric complications (101–103). Trophoblastic tumors often produce high levels of IL-8, G-CSF, and tumor-derived exosomes, which can strongly induce neutrophil NET formation (102). For example, in gastric cancer, Xia demonstrated that IL-8 acting through CXCR1/2 stimulates NETosis, which not only increases tumor cell capture by NETs and adhesion, but also enhances vascular permeability and cellular invasiveness, blocking NETs or IL-8 attenuates these effects (103). A similar mechanism seems to operate in hematogenously metastatic tumors like choriocarcinoma.

At the same time, GTN arises in an environment characterized by high levels of estrogen, progesterone, and human chorionic gonadotropin (hCG), together with regulatory immune subsets and local tolerogenic mechanisms (101–103). NET-associated histones and MPO, which are intrinsically cytotoxic, might therefore exert only limited anti-tumor effects; instead, NET-driven endothelial injury, complement activation, and platelet aggregation are likely to dominate, promoting aberrant angiogenesis and a high risk of thromboembolic events.

This interpretation is supported by studies of preeclampsia and other pregnancy complications, where placental syncytiotrophoblast microparticles and IL-8 strongly induce NET release in the intervillous space, leading to endothelial damage, heightened inflammation, and impaired placental perfusion (101), and NETs in maternal serum further activate complement and stimulate endothelial cells to secrete pro-inflammatory mediators (104). GTN may thus represent an extreme, malignant variant of this NET-driven vascular pathology, in which trophoblast-derived vesicles, pregnancy-like hormonal levels, and persistent NETosis converge to shape a highly pro-invasive and pro-thrombotic microenvironment distinct from non-gynecologic solid tumors.

### Role of NETs in vaginal reconstruction and repair

Vaginal reconstruction commonly results in significant tissue injury and fibrosis (105), particularly in patients with Mayer–Rokitansky–Küster–Hauser (MRKH) syndrome (106), often leading to vaginal stenosis, lifelong scarring, or even reoperation (105). Recent developments in biomaterial-based hydrogels have substantially improved wound healing and epithelial differentiation in neovaginal reconstruction. For instance, a kind of polydimethylsiloxane (PHS) hydrogel containing E2 provides three-dimensional structural support that enhances epithelial proliferation, collagen deposition, and closure of the defect, thereby restoring vaginal wall thickness and architecture to near-normal levels (107). In parallel, conductive hydrogels like EF@S-HGM can recruit neutrophils and trigger the formation of NETs, which sequester pathogens and release antimicrobial enzymes such as elastase and myeloperoxidase, thus reinforcing infection control and accelerating tissue repair (108). The resulting release of NE and MPO enhances pathogen clearance and accelerates tissue repair (108). However, unregulated or prolonged NET accumulation can drive persistent inflammation and collagen overproduction, exacerbating fibrosis.

Moreover, a healthy vaginal milieu is dominated by *Lactobacillus* spp., which maintain low pH, protect mucosal integrity, and suppress pathogens (38). In contrast, many neovaginas lack these commensals, predisposing them to dysbiosis, inflammation, and aberrant NETs activation (25). Thus, incorporating local *Lactobacillus* inoculation or probiotic regimens, potentially alongside NET-modulating agents, may help restore acidity, mitigate pathological NET release, re-establish immune equilibrium, and promote epithelial regeneration.

## Diagnostic, therapeutic, and endocrine-oriented future perspectives

NETs and their core components have emerged not only as byproducts of neutrophil activation but also as active contributors to the pathogenesis of multiple gynecologic diseases. Recent studies have increasingly identified NETs as both biomarkers and mediators in inflammatory and neoplastic conditions affecting the female reproductive tract. Elevated concentrations of NET-associated molecules, including cfDNA, citH3, NE, and MPO, which have been consistently detected in peripheral blood, menstrual effluent, and peritoneal fluid, where they correlate with inflammatory burden, disease stage, pelvic adhesions, and adverse prognosis (e.g., high cfDNA and NE levels in ovarian cancer, and increased citH3/MPO in severe endometriosis). Quantitative assessment of these components, therefore offers a promising adjunct to conventional imaging and histopathology, providing a non-invasive readout of disease activity and microenvironmental remodeling that can precede overt anatomical change (26, 43, 109). In gynecologic malignancies, rising NET activity in blood or tumor-adjacent fluid may herald the establishment of a pro-tumorigenic niche characterized by immunosuppression, angiogenesis, and ECM remodeling, whereas in benign inflammatory conditions such as endometriosis, dynamic NET signatures appear to track lesion initiation and adhesion formation.

A major conceptual advance emerging from recent study is that this NET biology is embedded within an endocrine landscape that differs fundamentally from that of other solid tumors. Reviews of female reproductive disorders, including ovarian, cervical, and endometrial cancer, endometriosis, and inflammation-related complications, have converged on a shared, hormone-sensitive inflammatory platform in which NETs operate (110). Experimental studies show that 17β-estradiol can enhance NETosis via membrane estrogen receptor GPER/GPR30, increasing PAD4 expression and histone citrullination (72), whereas progesterone, particularly in the pregnant milieu. Moreover, antagonizes estrogen and G-CSF-driven NET formation and reshapes neutrophil activation states (8, 81). NET enrichment in endometriosis lesions and menstrual effluent (45), further supports a model in which fluctuating estrogen and progesterone levels, reproductive events, and metabolic–endocrine states collectively tune NET programs in gynecologic tissues. Ovulation-induced G-CSF release and peritoneal neutrophil influx that facilitate NET-dependent peritoneal seeding of high-grade serous carcinoma (90), and obesity-related endocrine metabolic cues that intersect with NET formation in endometrial cancer and endometriosis (26, 71), underscore how endocrine physiology can be co-opted by tumors via NETs.

Building on this framework, several translational priorities emerge. First, prospective clinical studies should jointly profile circulating and tissue NET markers (cfDNA/dsDNA, citH3, MPO/NE, NET-related gene or lncRNA signatures) with detailed endocrine variables, menstrual phase, menopausal status, exogenous hormone use, ovarian reserve, and adiposity-related estrogen exposure—in patients with cervical, endometrial, and ovarian cancers as well as benign gynecologic disease. Such integrated phenotyping will be critical to test the hypothesis that estrogen-dominant states lower the threshold for NETosis in reproductive tissues, while progesterone or progestin therapy partially restrains NET formation and its pro-thrombotic, pro-metastatic consequences. Large, longitudinal cohorts incorporating these variables could establish standardized thresholds for diagnostic and prognostic use of NET panels and identify hormone-NET endotypes that stratify risk and therapeutic responsiveness (110).

Second, mechanistic models are needed to move from association to causality. *In vitro* co-cultures combining neutrophils with hormone-responsive epithelial or tumor cells under defined estradiol/progesterone conditions, as well as organoid–immune co-cultures and humanized mouse models, will allow direct quantification of how physiological and pharmacologic hormone levels reshape NET programs, DNA damage responses, and immune evasion in gynecologic tissues. Insights from endometriosis and preeclampsia, where neutrophil-derived NETs promote lesion implantation or placental vascular injury in hormonally regulated peritoneal and uteroplacental environments (45, 104), offer concrete entry points for these models. Parallel incorporation of spatial transcriptomics and multiplex imaging into gynecologic tumor specimens could map the spatial and temporal intersection of NETs with hormone receptors, stroma, vasculature, and immune subsets, refining where and when NET-blocking interventions are most likely to succeed.

Third, the therapeutic potential of the NET–sex hormone axis warrants systematic testing. Preclinical data already indicate that enzymatic degradation of NET structures using DNase I or pharmacological inhibition of NET formation via PAD4 inhibitors can reduce neutrophil-driven tissue injury, fibrotic remodeling, and epithelial damage in chronic gynecologic disease, and limit metastatic dissemination, immune suppression, and thrombotic events in gynecologic oncology models (109). Rational combinations of NET-targeting agents, like DNase I, PAD4 inhibitors, CXCR1/2 antagonists, or upstream cytokine blockade, with endocrine interventions (e.g., progestin-based regimens in endometrial cancer, hormonally informed timing of anti-NET therapy in ovarian cancer, or carefully tailored strategies in pregnancy-related disease) should be evaluated in biomarker-enriched cohorts characterized by high NET signatures and defined hormone profiles. Experience from preeclampsia and other NET-driven obstetric disorders, where pharmacological inhibition of NETosis is being actively explored (111, 112), provides a precedent for targeting NETs in hormonally complex settings.

## Conclusion

In summary, NETs have moved from being viewed as inert byproducts to central, measurable, and druggable mediators at the intersection of innate immunity, endocrine signaling, and tissue remodeling in the female reproductive tract. As the field moves toward clinical translation, two requirements are paramount: robust, standardized biomarker panels and rigorously designed trials. Integrating NET markers into high-throughput, multi-analyte diagnostic platforms, quantifying cfDNA/dsDNA, citH3, NE/MPO, and NET-related gene signatures, will be essential to define clinically meaningful thresholds for risk stratification and response assessment. Large prospective studies are needed to validate these panels for early diagnosis and longitudinal monitoring, and to test NET-targeted therapies such as DNase I and PAD4 inhibitors, alone or in rational combinations with chemotherapy, immunotherapy, anti-angiogenic agents, and endocrine regimens (109). In parallel, mechanistic work using human tissues, organoids, immune-competent organoid models, and cervicovaginal or endometrial co-cultures must clarify how NETosis interfaces with sex hormones, immune circuits, and the vaginal microbiome; such studies are crucial to refine indications, timing, and safety windows for intervention. If these biomarker-driven and mechanism-anchored strategies can be successfully integrated, NET-based approaches have the potential to deliver earlier detection, real-time monitoring, and more precise, often non-hormonal yet endocrine-aware therapeutic options, thereby opening a distinct translational avenue for improving outcomes and quality of life for women with gynecologic diseases.

## Glossary

NETs: Neutrophil extracellular traps
NE: Neutrophil elastase
MPO: Myeloperoxidase
VEGF: Vascular endothelial growth factor
NK cell: Natural killer cell
ROS: Reactive oxygen species
PGE₂: Prostaglandin E₂
MBL: Mannose-binding lectin
PMA: Phorbol 12-myristate 13-acetate
LPS: Lipopolysaccharide
C5a/C5b: Complement component 5a/5b
GM-CSF: Granulocyte-macrophage colony-stimulating factor
PAD4: Peptidylarginine deiminase 4
CTC: Circulating tumor cells
IL-8: Interleukin-8
IL-6: Interleukin-6
TNF-α: Tumor necrosis factor-alpha
MMP9: Matrix metallopeptidase 9
CXCL5: C-X-C motif chemokine ligand 5
NLRP3: NOD-, LRR- and pyrin domain-containing protein 3
GSDMD: Gasdermin D (caspase-1-dependent cleavage of gasdermin D)
N-GSDMD: N-terminal fragments of Gasdermin D
PD-1/PD-L1: Programmed cell death protein 1/Programmed death-ligand 1
NADPH: Nicotinamide Adenine Dinucleotide Phosphate
TLR2: Toll-Like Receptor 2
TLR4: Toll-Like Receptor 4
IL-1β: Interleukin-1 beta
IL-17: Interleukin-17
Th17: T helper 17 cell
CXCR2: C-X-C Motif Chemokine Receptor 2
HMGB1: High Mobility Group Box 1
VEGF: Vascular Endothelial Growth Factor
COX-2: Cyclooxygenase-2
RAGE: Receptor for Advanced Glycation End Products
NF-κB: Nuclear Factor Kappa-B
SOD: Superoxide Dismutase
TGF-β1: Transforming Growth Factor-beta 1
α-SMA: Alpha-Smooth Muscle Actin
CXCL8C-X-C: Motif Chemokine Ligand 8 (also known as IL-8)
